# Sex differences in perception of invisible facial expressions

**DOI:** 10.3389/fpsyg.2015.00392

**Published:** 2015-04-02

**Authors:** Sang Wook Hong, K. Lira Yoon, Sophia Peaco

**Affiliations:** ^1^Department of Psychology, Florida Atlantic University, Boca Raton, FL, USA; ^2^Department of Psychology, University of Notre Dame, Notre Dame, IN, USA

**Keywords:** sex differences, facial expressions, female advantage, continuous flash suppression (CFS), positivity bias

## Abstract

Previous research indicates that women are better at recognizing facial expressions than men. In the current study, we examined whether this female advantage in the processing of facial expressions also occurs at the unconscious level. In two studies, participants performed a simple detection task and a 4-AFC task while faces were rendered invisible by continuous flash suppression. When faces with full intensity expressions were suppressed, there was no significant sex difference in the time of breakup of suppression (Study 1). However, when suppressed faces depicted low intensity expressions, suppression broke up earlier in men than women, indicating that men may be more sensitive to facial features related to mild facial expressions (Study 2). The current findings suggest that the female advantage in processing of facial expressions is absent in unconscious processing of emotional information. The female advantage in facial expression processing may require conscious perception of faces.

## Introduction

Mounting evidence demonstrates sex differences in the processing of facial expressions. In particular, numerous studies suggest that women are superior in facial expression processing, broadly termed as the female advantage (see Hall, 1978; Kret and De Gelder, 2012, for review). For example, women recognize subtle facial emotions more accurately (Hoffmann et al., 2010) and use audio-visual, multisensory emotional information more efficiently (Collignon et al., 2010). This female advantage in facial expression processing is even present in young children: 3.5-year-old girls are as accurate as 5-year-old boys in facial expression recognition (Boyatzis et al., 1993).

The female advantage in facial expression processing seems to be more prominent in the speed, rather than the accuracy, of identifying facial expressions. For example, even studies that failed to find sex differences in recognition accuracy demonstrated faster identification of facial expressions in women than in men (Rahman et al., 2004; Vassallo et al., 2009). Other studies suggest that the female advantage may depend on the type of facial expressions. For example, women are better at recognizing facial expressions of fear and sadness (Mandal and Palchoudhury, 1985; Nowicki and Hartigan, 1988), while men are better at recognizing angry faces (Mandal and Palchoudhury, 1985; Wagner et al., 1986; Rotter and Rotter, 1988).

In the current study, we examined whether the sex differences in the processing of facial expressions stem from women’s enhanced unconscious processing of emotional information. Emotional information can be processed even when people are not consciously aware of the stimuli. For example, the amygdala actively responds to invisible fearful faces when a fearful face is rendered invisible by backward masking (e.g., Morris et al., 1999) and by interocular suppression (Pasley et al., 2004; Jiang and He, 2006). Research on an adaptation aftereffect also suggests that emotional information from faces can be processed without visual awareness. When the adapting face is suppressed from visual awareness, an adaptation aftereffect to facial identity is not observed (Moradi et al., 2005), but an adaptation aftereffect to facial expressions still occurs (Adams et al., 2010; Yang et al., 2010). In addition, emotional faces remain dominant for a longer period than neutral faces during conventional binocular rivalry (Alpers and Gerdes, 2007; Yoon et al., 2009), suggesting unconscious processing of facial expressions.

Is the female advantage in facial expression processing due to women’s enhanced unconscious processing of emotional information? To address this question, we assessed differences between men and women in their speed of detecting faces that were rendered invisible using continuous flash suppression (CFS). In CFS, one eye is presented with contour-rich, Mondrian-like patterns that continuously change, while the other eye is presented with a to-be-suppressed static image (Tsuchiya and Koch, 2005; Tsuchiya et al., 2006). The continuously flashing patterns prevent the stimulus presented to the other eye (i.e., facial pictures in the current study) from being consciously perceived for an extended period of time. Stronger or meaningful stimuli break up suppression sooner and can be consciously perceived earlier by the viewers in CFS, reflecting enhanced unconscious processing of the stronger stimuli. For example, upright faces break up suppression earlier than upside-down faces, and recognizable words break up suppression earlier than unrecognizable words (Jiang et al., 2007). In addition, emotional faces achieve perceptual awareness more quickly than neutral faces in CFS (Yang et al., 2007; Stein and Sterzer, 2012).

Continuous flash suppression is particularly advantageous for the purpose of the current study compared with other psychophysical techniques that can dissociate awareness and stimulation, such as backward masking (Breitmeyer, 1984). In backward masking, the target (facial pictures in our case) can be presented only very briefly and has to be followed immediately by a mask. Thus, sustained periods of invisibility are not possible when backward masking is used. Furthermore, participants can be aware of the presence of a masked target without being aware of what it was (Ogmen et al., 2003). Thus, the distinction between awareness and unawareness can be blurred in backward masking. In binocular rivalry, the duration and timing of suppression are difficult to control due to its stochastic nature. In addition, there are periods of mixed dominance in which participants perceive mixtures of both rival stimuli. In contrast to these approaches, a stimulus can be suppressed from visual awareness for several seconds at a time, with a strong initial suppression on every trial in CFS (Tsuchiya and Koch, 2005). We hypothesized that the female advantage would be present in unconscious processing of facial expressions. More specifically, we hypothesized that female participants would detect the invisible emotional faces faster than male participants [i.e., shorter response times (RTs) in women than men], indicating enhanced unconscious processing of facial expressions in women (vs. men).

## Study 1

Study 1 examined sex differences in unconscious processing of full-blown facial expressions. Specifically, we expected women (vs. men) to exhibit shorter RTs (i.e., a quicker breakup of suppression), suggesting the presence of the female advantage in unconscious processing of facial expressions. In addition, we were interested in the effects of (1) emotional valence (happy or angry) and (2) the sex of a face on the breakup of suppression. However, we did not have specific hypotheses regarding the effects of the type of facial expressions and the model’s sex. We employed a simple detection paradigm (Study 1A) and a 4-AFC paradigm (Study 1B) to ensure that results are not confined to a specific experimental paradigm.

### Method

#### Participants

Undergraduate students participated in exchange for course credit. Participants whose error rates were higher than 5% were excluded from all analyses (five male and three female). Consequently, data from 42 participants (21 male and 21 female) for each experiment were analyzed. All participants provided informed consent, and all procedures were approved by the Florida Atlantic University’s Institutional Review Board.

#### Apparatus and Stimuli

**FIGURE 1:**
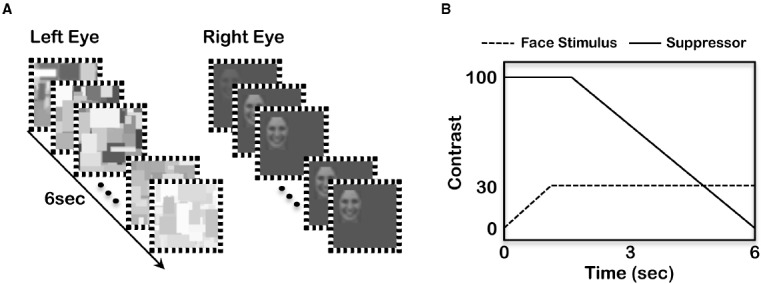
**(A)** Schematic diagram of stimulus presentation. **(B)** Changes in the contrast of the suppressor (solid line) and the face stimulus (dashed line).

Stimuli were presented on a Sony CPD-G520, 21-inch CRT display (100 Hz frame rate). The presentation of the stimuli and the collection of behavioral responses were controlled by the Psychophysics Toolbox (Brainard, 1997; Pelli, 1997) operating in Matlab (Mathworks). Stimuli were presented to participants positioned 90 cm from the CRT monitor whose luminance had been linearized from “black” (0.5 cd/m^2^) to “white” (70 cd/m^2^). A four-mirror stereoscope was used to present stimuli binocularly. In each trial, dynamically changing Mondrian-like patterns were presented within a 4° × 4° (visual angle) square aperture (with 4.25° × 4.25° fusion contour) to one randomly chosen eye, and a facial picture was presented to the other eye (see Figure 1A).

Four female and four male faces^1^ displaying angry, happy, and neutral expressions were chosen from the Karolinska Directed Emotional Faces (KDEF; Lundqvist et al., 1998). Each facial picture was resized to 1.45° × 2° visual angle. All facial pictures were adjusted to set identical root-mean-square (RMS) contrast to avoid breakup of suppression at different rates due to differences in physical contrast.

#### Tasks

***Detection task (Study 1A)***

Each trial began by pressing the spacebar on a keyboard. The to-be-suppressed facial pictures were presented at a random location within the 4° × 4° square aperture presented at the center of the view. During the first second of stimulus presentation, the overall contrast of a face was increased from 0 to its maximum contrast (30% RMS contrast) to prevent breakup of suppression due to transient signal caused by abrupt presentation (see Figure 1B). During the last 5 s, the contrast of facial pictures remained constant. The contrast of the suppressor (i.e., dynamically changing patterns) was fixed at their full contrast (100%) for the first 2 s and slowly ramped down to 0 over the following 4 s. Participants’ task was to indicate when they became aware of a face or any parts of a face during CFS, as quickly and accurately as possible by pressing the zero (0) key. The RTs, which indicated how long it took for the faces to break up suppression and to be detected, were recorded for analyses. If the participants did not perceive a face, they were instructed not to press any keys. Four facial pictures for each of three emotions were presented 12 times in random order and to a randomly selected eye, resulting in a total of 144 critical trials. In addition, there were 48 catch trials in which no facial picture was presented. If participants reported perceiving a face on these catch trials, it was considered as an error and used to calculate error rates.

***4-AFC task (Study 1B)***

The 4-AFC task was identical to the detection task with two exceptions. First, faces were presented in one of the four quadrants of the 4° × 4° square aperture (top-left, top-right, bottom-left, and bottom-right). The participants’ task was to report which quadrant a facial picture appeared as quickly and accurately as possible using the pre-designated keys. Each facial picture was presented 16 times (twice for each of four locations for each of two eyes), resulting in a total of 192 trials. Second, there was no catch trial, and error rates were calculated based on the number of trials in which participants indicated the location of a picture incorrectly.

### Results and Discussion

Response times were subjected to analyses of variance (ANOVAs) with one between-subject factor (i.e., participants’ sex) and two within-subject factors (i.e., models’ sex and facial expressions). Incorrect trials and detection task trials in which participants did not press the key (less than 3% of the data) were excluded from all the analyses. The means and standard errors (SEs) are presented in Table 1.

**TABLE 1:** **Means (SEs) for the breakup of suppression as a function of model’s sex and facial expressions for the detection (Study 1A) and the 4-AFC (Study 1B) tasks with full-intensity facial expressions**.

Expression	Model sex	Detection task		4-AFC task	
		Mean (second)	SE (second)	SE (second)	SE (second)
Happy	Female	2.843	0.112	2.146	0.082
	Male	2.952	0.119	2.372	0.100
Angry	Female	3.030	0.130	2.357	0.093
	Male	3.352	0.134	2.719	0.117
Neutral	Female	2.869	0.111	2.246	0.087
	Male	3.207	0.131	2.434	0.096

### Detection Task (Study 1A)

**FIGURE 2:**
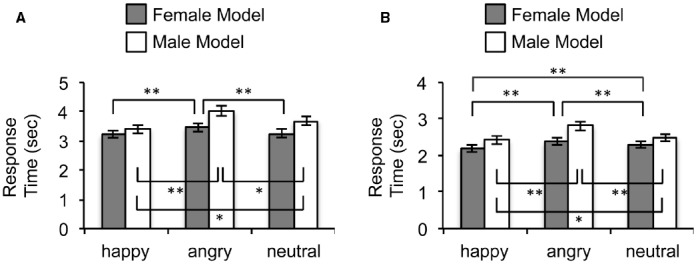
**Results of Study 1. (A)** RTs for female and male faces as a function of emotional valence in the detection task. **(B)** RTs for female and male faces as a function of emotional valence in the 4-AFC task (*p < 0.05; **p < 0.01).

The main effect for participants’ sex was not significant [*F*(1,40) = 0.185, *p* = 0.669, ${\text{η}}_{\text{p}}^{\text{2}}$ = 0.005], indicating that the processing of facial expressions under CFS generally does not differ significantly between men and women. The main effects for models’ sex [*F*(1,40) = 49.12, *p* < 0.001, ${\text{η}}_{\text{p}}^{\text{2}}$ = 0.55] and facial expressions [*F*(2,80) = 16.53, *p* < 0.001, ${\text{η}}_{\text{p}}^{\text{2}}$ = 0.29] were significant. These main effects were qualified by a significant models’ sex × facial expressions interaction [*F*(2,80) = 3.84, *p* = 0.026, ${\text{η}}_{\text{p}}^{\text{2}}$ = 0.09], which is depicted in Figure 2A. For pictures posed by male models, the main effect of facial expression was significant [*F*(2,82) = 13.83, *p* < 0.001, ${\text{η}}_{\text{p}}^{\text{2}}$ = 0.252]. Follow-up tests revealed that happy faces broke up suppression faster than both neutral [*t*(41) = 3.82, *p* < 0.001] and angry [*t*(41) = 4.42, *p* < 0.001] faces, whereas angry faces broke up suppression slower than neutral faces [*t*(41) = 2.02, *p* = 0.05]. The main effect of facial expression was also significant for pictures posed by female models [*F*(1,40) = 5.60, *p* = 0.005, ${\text{η}}_{\text{p}}^{\text{2}}$ = 0.12]. Similar to the male pictures, happy faces broke up suppression faster than angry faces [*t*(41) = 3.02, *p* = 0.004], whereas angry faces broke up suppression slower than neutral faces [*t*(41) = 2.69, *p* = 0.01]. However, the RTs for happy faces were not significantly different from neutral faces [*t*(41) = 0.43, *p* = 0.67] when posed by female models. Lastly, the female faces broke up suppression more quickly than the male faces for all three facial expressions (all *t*s ≥ 2.60, all *p*s < 0.013).

### 4-AFC Task (Study 1B)

Again, the main effect for participants’ sex was not significant [*F*(1,40) = 0.013, *p* = 0.911, ${\text{η}}_{\text{p}}^{\text{2}}$ < 0.001]. The main effects for models’ sex [*F*(1,40) = 95.44, *p* < 0.001, ${\text{η}}_{\text{p}}^{\text{2}}$ = 0.70] and facial expressions were significant [*F*(2,80) = 57.20, *p* < 0.001, ${\text{η}}_{\text{p}}^{\text{2}}$ = 0.59]. These main effects were qualified by a significant models’ sex × facial expressions interaction [*F*(2,80) = 8.04, *p* = 0.001, ${\text{η}}_{\text{p}}^{\text{2}}$ = 0.17], which is depicted in Figure 2B. The main effects of facial expressions were significant for both the male [*F*(1,40) = 39.39, *p* < 0.001, ${\text{η}}_{\text{p}}^{\text{2}}$ = 0.490] and the female [*F*(1,40) = 25.62, *p* < 0.001, ${\text{η}}_{\text{p}}^{\text{2}}$ = 0.385] faces. Follow-up tests revealed that the RTs for happy faces were significantly faster than neutral faces, and angry faces were significantly slower than neutral faces to break up suppression for both the female and the male faces (all *t*s ≥ 2.12, all *p*s < 0.04). Replicating the findings from Study 1A, the female faces broke up suppression more quickly than the male faces for all three facial expressions (all *t*s ≥ 5.25, all *p*s < 0.001).

Overall, the findings revealed that emotional expressions significantly affect the time it takes for faces to access visual awareness. These findings are consistent with previous studies demonstrating that emotional information is indeed processed even when faces are not consciously perceived (Morris et al., 1999; Pasley et al., 2004; Jiang and He, 2006; Yang et al., 2007). Inconsistent with our hypothesis, the detection times did not differ between the male and the female participants. Thus, our results suggest that the female advantage in facial expression processing may require conscious recognition of facial expressions. Interestingly, the female faces broke up suppression more quickly than the male faces. These results suggest that facial features that differentiate female and male faces can be processed during CFS. More studies are required to understand specific mechanisms associated with faster access to visual awareness for female (vs. male) faces.

## Study 2

We found no significant sex differences in the breakup of suppression in Study 1, which suggests that the female advantage in the processing of facial expressions may not be present at the unconscious level. However, the use of full-blown expressions in Study 1 could have masked any sex differences due to ceiling effects. For example, the female advantage in facial emotion recognition was observed for subtle facial expressions (i.e., 50%) but not for full-blown facial expressions (Hoffmann et al., 2010). Therefore, we examined sex differences in unconscious processing of facial expressions using low intensity (50%) facial expressions in Study 2. Emotional intensity of 50% was selected given that the female advantage was still present at this intensity when presented consciously. We expected that women (vs. men) would exhibit quicker breakup of suppression.

### Method

#### Participants

Undergraduate students participated in exchange for course credit. Participants whose error rates were higher than 5% were excluded from analyses (four male and six female), and consequently data from 40 participants (20 male and 20 female) for each experiment were analyzed. All participants provided informed consent, and all procedures were approved by the Florida Atlantic University’s Institutional Review Board.

#### Stimuli

Happy and angry faces with the intensity of 50% (i.e., a 50:50 blend of neutral and emotional faces) were used. The face stimuli with 50% emotional intensity were created by morphing a neutral and a full-blown emotional face (either an angry or a happy face) using image-morphing software (Fantamorph). The KDEF pictures (Lundqvist et al., 1998) that were used in Study 1 were used to create the stimuli.

#### Tasks

Experimental tasks used in Study 2 were identical to the ones used in Study 1 except for two changes. First, full intensity facial expressions were used in Study 1, whereas half intensity expressions were used in Study 2. Second, neutral expression was not included in Study 2. In the detection task (*Study 2A*), four facial pictures for happy and angry emotions were presented 12 times, resulting in a total of 96 critical trials. Forty-eight catch trials were included in Study 2A. In the 4-AFC task (Study 2B), each facial picture was presented 16 times (twice for each of four locations for each of two eyes), resulting in a total of 128 trials.

### Results and Discussion

Like Study 1, Participant Sex × Model Sex × Emotion ANOVAs were conducted with RTs as dependent variables (DVs). Incorrect trials and detection task trials in which participants did not press the key (less than 3% of the data) were excluded from all the analyses. The means (SEs) are presented in Table 2.

**TABLE 2:** **Means (SEs) for the breakup of suppression as a function of model’s sex and facial expressions for the detection (Study 2A) and the 4-AFC (Study 2B) tasks with half-intensity facial expressions**.

Expression	Model sex	Detection task		4-AFC task	
		Mean (second)	SE (second)	Mean (second)	SE (second)
Happy	Female	2.775	0.116	2.248	0.107
	Male	2.979	0.128	2.500	0.122
Angry	Female	2.861	0.125	2.343	0.107
	Male	3.270	0.144	2.582	0.123

#### Detection Task (Study 2A)

**FIGURE 3:**
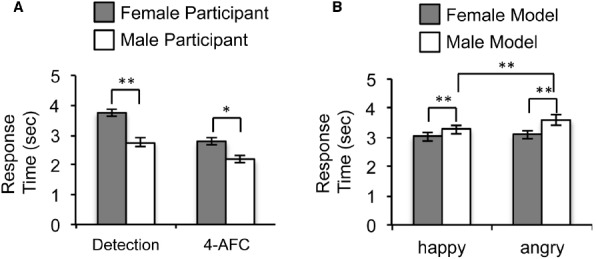
**Results of Study 2. (A)** RTs for female and male participants in the detection task (left) and in the 4-AFC task (right). RTs for happy and angry faces were collapsed together given the absence of significant interaction between participants’ sex and facial expression. **(B)** RTs for female and male faces as a function of facial expressions in the detection task (*p < 0.05; **p < 0.01).

Unlike Study 1, the main effect for participants’ sex was significant [*F*(1,38) = 11.38, *p* = 0.002, ${\text{η}}_{\text{p}}^{\text{2}}$ = 0.23]. Inconsistent with the notion of the female advantage, men became aware of the faces earlier than women (Figure 3A). Consistent with findings from Study 1, the main effects for models’ sex [*F*(1,38) = 41.95, *p* < 0.001, ${\text{η}}_{\text{p}}^{\text{2}}$ = 0.53] and facial expressions [*F*(1,38) = 23.55, *p* < 0.001, ${\text{η}}_{\text{p}}^{\text{2}}$ = 0.383] were significant. These main effects were again qualified by a significant interaction between models’ sex and facial expressions, *F*(1,38) = 6.63, *p* = 0.014, ${\text{η}}_{\text{p}}^{\text{2}}$ = 0.15 (Figure 3B). There was no significant difference in RTs between happy and angry faces for the female models [*t*(39) = 1.47, *p* = 0.15], but the difference between happy and angry faces was significant when posed by the male models [*t*(39) = 5.54, *p* < 0.001]. For both angry and happy faces, the female pictures broke up suppression earlier than the male pictures (both *t*s ≥ 3.93 and *p*s < 0.001).

#### 4-AFC Task (Study 2B)

Replicating findings from Study 2A, the main effect for participants’ sex was significant [*F*(1,38) = 6.36, *p* = 0.013, ${\text{η}}_{\text{p}}^{\text{2}}$ = 0.143], suggesting sex differences in unconscious processing of facial expressions. Again, men became aware of the faces earlier than women. In addition, the main effect for models’ sex [*F*(1,38) = 67.38, *p* < 0.001, ${\text{η}}_{\text{p}}^{\text{2}}$ = 0.64] was significant, with the female faces breaking up suppression more quickly than the male faces. The main effect for facial expressions was also significant [*F*(2,38) = 10.70, *p* = 0.002, ${\text{η}}_{\text{p}}^{\text{2}}$ = 0.22], with the happy faces breaking up suppression more quickly than the angry faces. Unlike Study 1 and Study 2A, the interaction between models’ sex and facial expressions was not significant [*F*(1,38) = 0.087, *p* = 0.77, ${\text{η}}_{\text{p}}^{\text{2}}$ = 0.002].

## Discussion

Current findings stand in contrast to previous studies demonstrating the female advantage in recognizing facial expressions (Hall, 1978; Campbell et al., 2002; Hampson et al., 2006; Collignon et al., 2010). Specifically, there were no sex differences in perceiving full-blown facial expressions that had been visually suppressed. When faces with mild expressions were visually suppressed, there were sex differences. Contrast to our hypothesis, it took less time for men (vs. women) to perceive emotional facial pictures that had been visually suppressed. Thus, current findings suggest that the female advantage in the recognition of facial expressions may not be present in unconscious processing of emotional information.

### Implication to the Female Advantage in Processing of Facial Expressions

Differences in tasks might be responsible for the discrepancy between previous studies reporting the female advantage and the current study. Studies supporting the female advantage generally asked participants to explicitly judge or discriminate facial expressions (Rotter and Rotter, 1988; Hall and Matsumoto, 2004; Rahman et al., 2004; Collignon et al., 2010). On the other hand, participants in the current study performed detection tasks without any judgment of facial expressions. Furthermore, the use of CFS in the current study could have prevented any explicit recognition of facial expressions. Although CFS may allow unconscious processing of emotional information (Jiang and He, 2006; Yang et al., 2007; Stein and Sterzer, 2012), it still limits conscious recognition of facial expressions (Adams et al., 2010; Yang et al., 2010). Thus, previously reported female advantage in the processing of facial expressions may result from women’s better ability to label the consciously perceived facial features, and not because women have greater perceptual sensitivity to emotional faces.

Alternatively, our finding that men broke up suppression earlier than women might reflect men’s greater motor preparedness when processing emotional stimuli. For example, men (vs. women) exhibited greater activation in the premotor cortex while viewing threatening stimuli, even though men and women did not differ in their recognition of emotional stimuli (Kret et al., 2011). Thus, men may act faster (i.e., press the response keys quicker) than women due to efficient action preparedness, but not necessarily due to more efficient processing of emotional stimuli. However, two critical questions should be answered before attributing the current findings to men’s greater action preparedness. First, can emotional stimuli activate the premotor cortex when the stimuli are suppressed from visual awareness? Second, do positive stimuli also induce a greater premotor cortex activity in men than women?

In the current study, men detected suppressed faces quicker than women only when the expressions were of lower intensity (50%). Are men more sensitivity to facial features related to mild facial expressions? For simple visual stimuli such as sine-wave gratings, men and women do not differ in their contrast sensitivity (Solberg and Brown, 2002). Thus, men’s faster RTs in the current study cannot be due to higher sensitivity to low level features, such as high contrast around eyes and mouth. Further research is required to understand mechanisms underlying the sex differences in the processing of unconscious facial expressions.

### Positivity Bias in Facial Expression Processing

Considering the importance of detecting threatening stimuli for one’s survival, angry faces might be processed more efficiently. The notion of this anger superiority effect has been supported by studies demonstrating that angry faces are detected more efficiently than happy faces among a crowd of distractors (Fox et al., 2000; Öhman et al., 2001; Horstmann and Bauland, 2006). However, angry faces broke up suppression slower than happy faces in the current study. Although this finding is inconsistent with the anger superiority effect, there is growing evidence demonstrating the positivity bias. For example, happy faces are detected faster than angry faces in search tasks (Juth et al., 2005; Calvo and Nummenmaa, 2008) and in a single detection task (Grimshaw et al., 2004). Happy faces are also recognized faster than faces expressing negative emotions (Leppänen and Hietanen, 2004). It is worth noting that angry faces also broke up suppression slower than neutral faces in the current study. This result is puzzling because faces with negative emotion are predominantly perceived longer than neutral faces during binocular rivalry, indicating that negative faces are stronger stimuli compared to neutral faces (Yoon et al., 2009). However, slower responses to negative (vs. neutral) stimuli have been reported in attentional tasks (Fox et al., 2001), which have been posited as a result of delayed attentional disengagement. Because the influence of attention on the interocularly suppressed stimuli is not well understood, more research is required to address whether delayed attentional disengagement underlies the slower breakup of suppression for angry faces.

It remains unclear whether the emotional content *per se* affects the detection of faces that are suppressed from visual awareness. Although the current study cannot directly address this issue, previous research provides some clues. Fearful (vs. neutral and happy) faces broke up suppression more quickly when only the eye region was presented instead of a full facial picture, suggesting that a specific facial feature (i.e., higher contrast around eyes) is responsible for the early breakup of suppression for fearful faces (Yang et al., 2007). Consistent with the current findings, Stein and Sterzer (2012) demonstrated that schematic happy faces broke up suppression more quickly than angry faces. Similar to Yang et al. (2007) the orientation of mouth-contour contributed to the early breakup of suppression for happy faces. Thus, facial features and their specific configurations related to specific emotions, not emotional content *per se*, affect the speed in which a face is detected under CFS. On the other hand, given that the facial features (enlarged eyes for fear) and configurations (particular orientation of mouth-contour for happy and angry) are the primary carrier of emotional information (Whalen et al., 2004; Leppänen and Hietanen, 2007), the influence of emotional content on the access to visual awareness cannot be excluded.

### Unconscious Processing of Sex of a Face

Pervious research has demonstrated that information regarding the sex of a face is not processed when the face is suppressed from visual awareness (e.g., Amihai et al., 2011). In the current study, however, the female faces broke up suppression quicker than the male faces, suggesting that information about the sex of a face may be processed even when a face is suppressed from visual awareness. Some previous findings indeed support this notion. For example, spatial filtration differentially affected the categorization of male and female faces (Cellerino et al., 2004), indicating that information about the sex of a face is processed even when faces are not consciously perceivable. Male angry faces presented for 34 ms were better recognized than female angry faces, suggesting that information about the sex of a face is processed relatively early (Goos and Silverman, 2002). Even during CFS, the facial sex adaptation effect can be recovered when spatial attention is directed to the location where the suppressed, adapting face is presented (Shin et al., 2009).

Given prior research demonstrating that women are more expressive than men (see Kret and De Gelder, 2012, for review), one could suspect that the female faces used in the current research express more intense expressions than the male faces, thereby allowing the female faces to break up suppression quicker than the male faces. This, however, was not the case. For the models used in the current study, recognition accuracies (Goeleven et al., 2008) for happy faces did not differ significantly between the male (96%) and the female (96.25%) faces. Furthermore, angry male faces (71.75%) were better recognized than female angry faces (57%). Thus, at least in the current study, the early breakup of suppression for female faces cannot be attributed to the difference in expressiveness between female and male models. Clearly more research is needed to better understand why people are more sensitive to female faces.

## Conclusion

Although the findings reported here are based on relatively small number of participants, the current study reveals three important aspects of social information processing by assessing the unconscious processing of faces. First, we found that men may be more sensitive to facial features related to mild facial expressions than women. This, in turn, suggests that the generally known female advantage in the recognition of facial expressions requires conscious perception of faces. Second, happy (vs. angry) faces are processed more efficiently, irrespective of a perceiver’s or a model’s sex. Lastly, the current study provides evidence that gender-related facial features could be processed even when facial pictures are suppressed from visual awareness.

### Conflict of Interest Statement

The authors declare that the research was conducted in the absence of any commercial or financial relationships that could be construed as a potential conflict of interest.

## Footnotes

1 The following faces from the KDEF (Lundqvist et al., 1998) were used in the study: Female: 1, 6, 11, and 33; Male: 1, 7, 28, and 29.
